# High tumor mutation burden is associated with DNA damage repair gene mutation in breast carcinomas

**DOI:** 10.1186/s13000-020-00971-7

**Published:** 2020-05-11

**Authors:** Ping Mei, C. Eric Freitag, Lai Wei, Yunxiang Zhang, Anil V. Parwani, Zaibo Li

**Affiliations:** 1 Department of Pathology, Guangdong Provincial People’s Hospital, Guangzhou, China; 2 Department of Pathology, Mayo Clinic, Rochester, MN USA; 3 Department of Biomedical Informatics, Center for Biostatistics, The Ohio State University, Columbus, OH USA; 4 Weifang People’s Hospital, Weifang, China; 5 Department of Pathology, The Ohio State University Wexner Medical Center, 410 W. 10th Ave, Columbus, 43210 OH USA

**Keywords:** Breast carcinoma, Genetic mutation, Tumor mutation burden, DNA damage repair genes

## Abstract

**Background:**

Immunotherapy has demonstrated encouraging clinical benefits in patients with advanced breast carcinomas and Programmed death ligand 1 (PD-L1) expression has been proposed as an immunotherapy biomarker. Challenges with current PD-L1 testing exist and tumor mutation burden (TMB) is emerging as a biomarker to predict clinical response to immunotherapy in melanoma and non-small cell lung cancer patients. However, TMB has not been well characterized in breast carcinomas.

**Methods:**

The study cohort included 62 advanced breast cancer patients (13 primary and 49 metastatic). Genetic alterations and TMB were determined by FoundationOne CDx next generation sequencing (NGS) and the association with clinicopathologic features was analyzed.

**Results:**

High TMB was observed in a relatively low frequency (3/62, 4.8%). TMB levels were positively associated tumor infiltrating lymphocytes and significantly higher TMB was observed in breast carcinomas with DNA damage repair gene mutation(s). There was no significant association between TMB levels and other analyzed clinicopathologic characteristics.

**Conclusions:**

Our data indicate the importance of DNA damage repair proteins in maintaining DNA integrity and immune reaction and breast carcinoma patients with DDR mutation may benefit from immunotherapy.

## Introduction

Breast cancer (BC) is the most common malignancy in women [1] and biomarkers including estrogen receptor (ER)s, progesterone receptor (PR), and human epidermal growth factor receptor 2 (HER2), are routinely performed for therapeutic decision-making [2]. Although anti-hormonal and anti-HER2 targeted therapies are available for a large percentage of BC patients, up to 30% BC patients develop advanced disease during their disease courses [3, 4]. The lack/loss of efficacy of targeted therapies and the relatively poor prognosis of advanced BC patients have created the need to evaluate novel treatment approaches, including immunotherapy [5–7]. Recent studies have demonstrated pembrolizumab and atezolizumab plus nab-paclitaxel have demonstrated encouraging clinical benefits in patients with advanced triple negative BC [5, 6]. Although Programmed death ligand 1 (PD-L1) expression has been proposed as a biomarker for immunotherapy, challenges with PD-L1 testing exist, including interassay variability among different PD-L1 immunohistochemistry (IHC) assays with different reagents and platforms, lack of standardization among different PD-L1 IHC assays, and even interobserver variability in interpreting PD-L1 IHCs [8–12].

Tumor mutation burden (TMB) is defined as the total number of somatic mutations in a region of tumor genome and is associated with the immunogenicity of many different tumors, including BC [13, 14]. TMB is emerging as potential biomarker for immunotherapy decisions in melanoma or non-small lung cancer patients [15–23]; however, TMB has not been well characterized in BCs. Whole exome sequencing is the standard method to determine TMB, but it is time consuming and too expensive for routine clinical practice. Recently, commercially available cancer gene panels, such as FoundationOne CDx, have been shown to have similar accuracy in determining TMB and similar ability to predict outcomes to immunotherapy [13, 21, 24, 25]. In this study, we aimed to investigate TMB in BCs using FoundationOne CDx NGS and its association with different clinicopathologic features including histologic types, hormone receptor and HER2 status, and different genetic mutations.

## Methods

### Patients and specimens

The study cohort included 62 advanced breast cancer patients (13 primary and 49 metastatic) within a study period between January 2014 and June 2018. The specimens from these patients were sent to Foundation Medicine (Cambridge, MA) for analyzing genetic alterations and TMB by FoundationOne CDx next generation sequencing (NGS) (Foundation Medicine, Cambridge, MA). This study was approved by the Ohio State University institutional Research Board. Clinicopathologic characteristics were collected and breast cancer biomarkers (ER, PR, and HER2) were evaluated by breast subspecialized pathologists, with manual quantification [ER: clone SP1 (Spring, Pleasanton, CA), PR: clone PgR 636 (DAKO, Carpinteria, CA), HER2: 4B5 clone (Ventana, Tucson, AZ)].

### Analyzing genetic alterations and tumor mutation burden by FoundationOne CDx NGS

Genetic alterations were determined by FoundationOne CDx NGS performed at Foundation Medicine and the results were extracted from FoundationOne CDx reports. TMB was also determined by FoundationOne CDx as the number of somatic, coding base substitutions and short insertions and deletions per megabase of tumor genome examined [13]. TMB levels are divided into three groups on FoundationOne CDx reports, including low TMB (1–5 muts/mb), intermediate TMB (6–19 muts/mb), and high TMB (≥ 20 muts/mb).

### Evaluating tumor infiltrating lymphocytes (TILs)

At Ohio State University, all slides from tumor cases had been digitally scanned (Philips Intel-liSite). Representative digital slides from our cohort except 5 cytology cases were reviewed with the Philips Digital Pathology Solutions viewer and TILs were scored as a percentage by reviewing the slides at 50x, 100x, and 200x magnifications.

### DNA damage repair gene mutation analysis

Thirty-four DNA damage repair (DDR) genes are included in FoundationOne CDx NGS and analyzed for genetic alterations. These genes belongs several DDR canonical pathways including nucleotide excision repair (NER), mismatch repair (MMR), Fanconi Anemia (FA), homologous recombination (HR), checkpoint and others [26]. (Table 1).

**Table 1:** DNA damage repair genes included in FoundationOne CDx NGS panel

Pathways	NER	MMR	FA	HR	Checkpoint	Others
Genes	ERCC2	MLH1	BRCA2	BRCA1	ATM	POLE
	ERCC3	MSH2	BRIP1	MRE11A	ATR	MUTYH
	ERCC4	MSH6	FANCA	NBN	CHEK1	PARP1
	ERCC5	PMS1	FANCC	RAD50	CHEK2	RECQL4
		PMS2	PALB2	RAD51	MDC1	
			RAD51C	RAD51B		
			BLM	RAD51D		
				RAD52		
				RAD54L		

Abbreviations: *NER* nucleotide excision repair, *MMR* mismatch repair, *FA* Fanconi Anemia, *HR* homologous recombination

### Statistical analysis

All clinicopathologic variables were summarized using percentages and descriptive statistics (mean, range, frequencies). T test was used to compare the continuous values among different groups. Statistics were performed using SAS version 9.3 (SAS Institute Inc., Cary, North Carolina). For all results, a *P*-value of < 0.05 was considered significant.

## Results

### The demographic features of study cohort

**Fig. 1:**
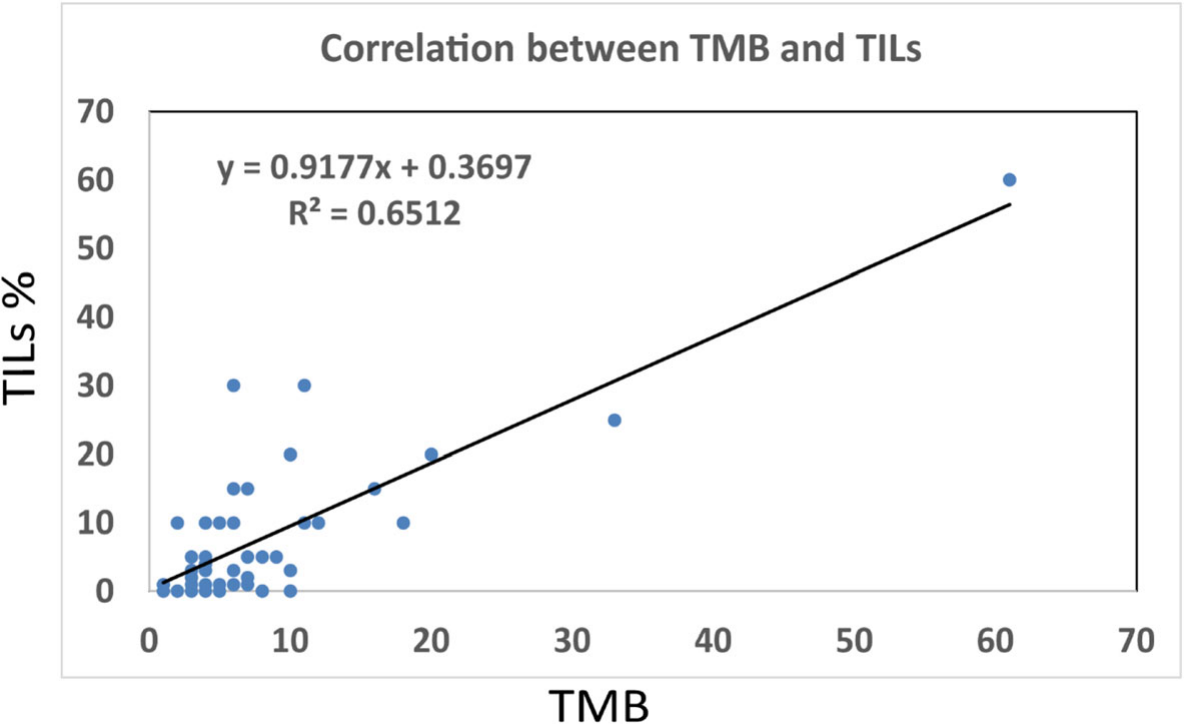
Correlation between tumor mutation burden (TMB) and tumor infiltrating lymphocytes (TILs). The Pearson correlation coefficient (*r*) for TMB and TILs was 0.80696 (*n* = 57; *P* < .0001). (y = 0.9177x + 0.3697, R^2^ = 0.6512)

The average age of the patient’s studied in this cohort was 53.8 years old (range 30–78). The majority of specimens were from metastatic locations (49, 79.0%) because FoundationOne CDx was ordered mostly in patients with advanced stage disease. Fifty-two (83.9%) cases were invasive ductal carcinoma (IDC), 6 (9.7%) were invasive lobular carcinoma, 2 (3.2%) were metaplastic carcinoma, and 2 (3.2%) were neuroendocrine carcinomas. As for breast cancer biomarkers, 36 (58.1%) were ER positive, 38 (61.3%) were PR positive, 5 (8.1%) were HER2 positive, and 22 (35.5%) were triple negative. Among 62 cases, 3 (4.8%) had high TMB, 27 (43.6%) had intermediate TMB, and the remaining 32 (51.6%) had low TMB. Due to the rarity of high TMB, cases with high and intermediate TMB were grouped together and compared with cases with low TMB regarding to their clinicopathologic features. The group of cases with intermediate or high TMB showed significantly increased TILs than the group of cases with low TMB (*p* = 0.0018). In addition, a moderate correlation between TMB and TILs was identified by the Pearson correlation analysis with a coefficient (*r*) of 0.80696 (*n* = 57, *P* < .0001, y = 0.9177x + 0.3697, R^2^ = 0.6512). (Fig. 1) There was no significant association between TMB levels and other analyzed clinicopathologic characteristics, including biomarker status, histologic types and tumor nuclear grade. (Table 2).

**Table 2:** Demographic characteristics and tumor mutation burdens of the study cohort (*n* = 62)

		Total		TMB high/intermediate		TMB low		*p* value
Case #		62		30		32		
Age		53.8	30–78	54.6	31–74	53.1	30–78	NS
Location	Primary	13	21.0%	6	20.0%	7	21.9%	NS
	Metastatic	49	79.0%	24	80.0%	25	78.1%	NS
Biomarkers	ER-positive	36	58.1%	19	63.3%	17	53.1%	NS
	PR-positive	18	29.0%	9	30.0%	9	28.1%	NS
	HER2-positive	5	8.1%	2	6.7%	3	9.4%	NS
	Triple-negative	22	35.5%	8	26.7%	14	43.8%	NS
Histologic type	Ductal, NOS	52	83.9%	26	86.7%	26	81.3%	NS
	Lobular	6	9.7%	3	10.0%	3	9.4%	NS
	Metaplastic	2	3.2%	1	3.3%	1	3.1%	NS
	Neuroendocrine	2	3.2%	0	0.0%	2	6.3%	NS
Gene mutation	*p53*	37	59.7%	20	66.7%	17	53.1%	NS
	*PIK3CA*	21	33.9%	9	30.0%	12	37.5%	NS
	*BRCA (1/2)*	6	9.7%	5	16.7%	1	3.1%	0.0002
Nuclear grade		2.5	2–3	2.5	2–3	2.4	2–3	NS
Tumor infiltrating lymphocytes		7.1%	0–60%	11.4%	0–60%	3.5%	0–10%	0.0018

Abbreviations: *TMB* tumor mutation burden, *ER* estrogen receptor, *PR* progesterone receptor

The most common gene mutation identified among 62 cases was *TP53* (59.7%) followed by *PIK3CA* (33.9%). Interestingly, of the 6 BCs with *BRCA* (1/2) mutations analyzed, 5 of them had intermediate or high TMB, while only one case showed low TMB (*p* = 0.0002). (Table 2) The association between TMB and DNA damage repair pathway.

Thirty-four DDR genes are included in FoundationOne CDx NGS panel and analyzed for genetic alterations. Thirteen cases showed at least one DDR gene mutation and the remaining 49 cases did not show any DDR gene mutation. Clinicopathologic features and TMB were analyzed between DDR mutated and non-DDR mutated cases. BCs harboring DDR mutation(s) averaged a higher TMB compared to those without DDR mutation (12.08 average mutations vs. 6.57; *p* = 0.043). No significant difference was found in other analyzed clinicopathologic characteristics between DDR mutated and non-DDR mutated cases. (Table 3).

**Table 3:** Tumor mutation burden between DDR-mutated BCs and non-DDR-mutated BCs

		DDR mutated		Non-DDR mutated		Total		p Value
		# (average)	% (range)	# (average)	% (range)	# (average)	% (range)	
#		13		49		62		
Age		50.7	36–70	54.6	30–78	53.8	30–78	0.2718
Specimens	Biopsy	10	76.9%	37	75.5%	47	75.8%	
	Excision	1	7.7%	9	18.4%	10	16.1%	
	Cytology	2	15.4%	3	6.1%	5	8.1%	
Locations	primary	3	23.1%	10	20.4%	13	21.0%	NS
	Metastatic	10	76.9%	39	79.6%	49	79.0%	
Biomarkers	ER/PR+	10	76.9%	25	51.0%	35	56.5%	0.01769
	HER2+	2	15.4%	3	6.1%	5	8.1%	
	TNBC	1	7.7%	21	42.9%	22	35.5%	
Histologic types	Ductal, NOS	11	84.6%	41	83.7%	52	83.9%	NS
	Lobular	1	7.7%	5	10.2%	6	9.7%	
	Metaplastic	1	7.7%	1	2.0%	2	3.2%	
	Neuroendocrine	0	0.0%	2	4.1%	2	3.2%	
TMB		12.08	4–33	6.57	1–61	7.73	1–61	0.042909
Nuclear grade		2.5	2–3	2.4	2–3	2.5	2–3	NS
Tumor infiltrating lymphocytes		8.8%	1–25%	6.6%	0–60%	7.1%	0–60%	NS

Abbreviations: *DDR* DNA damage repair, *ER* estrogen receptor, *PR* progesterone receptor, *TNBC* triple negative breast cancer, *TMB* tumor mutation burden

### Cases with high TMB (≥ 20) harbored either *MAGI2* or *PTEN* genetic mutations

**Fig. 2:**
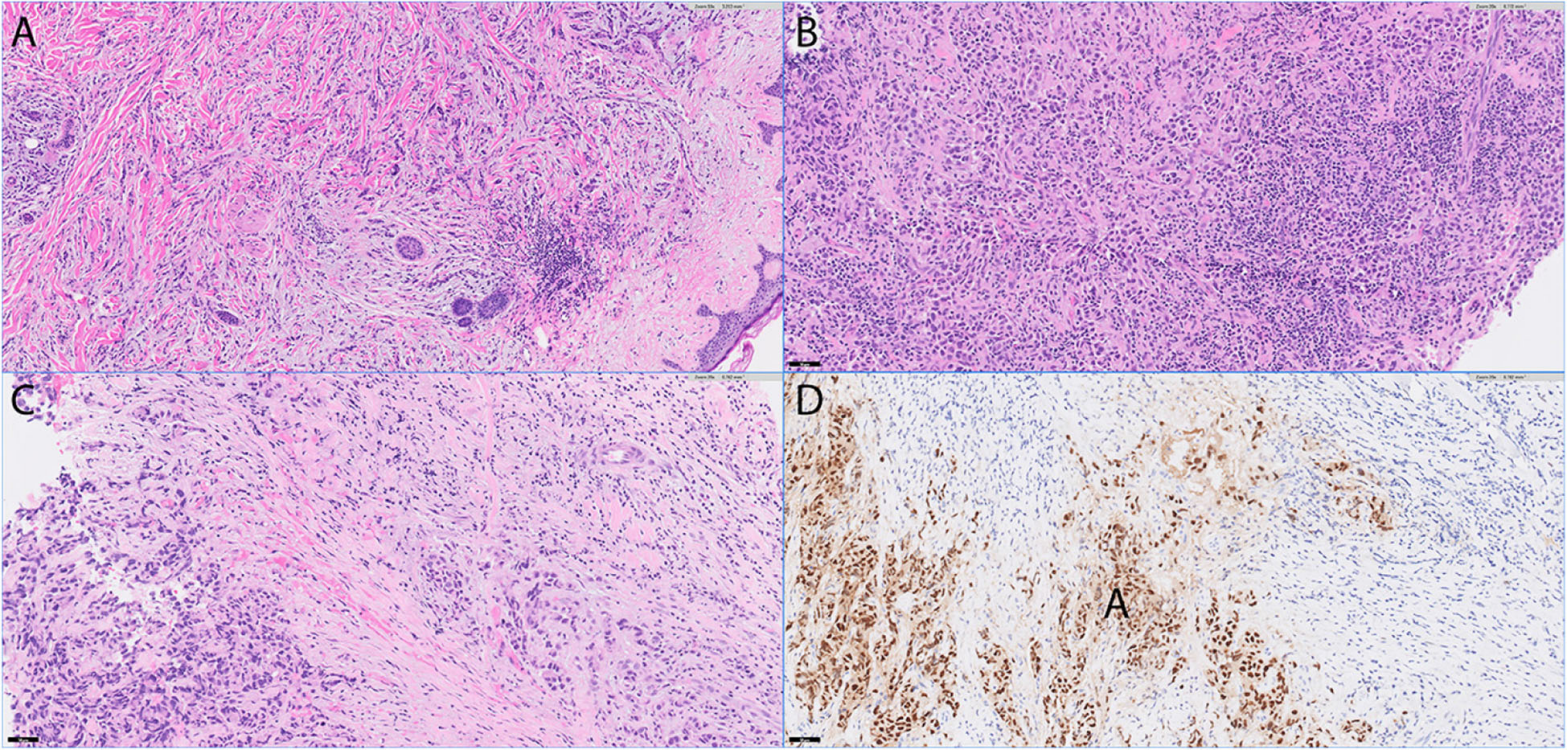
Three cases with high tumor mutation burden. **a**-**c** Representative H&E images from three cases (#1–3) with high tumor mutation burden. **d** Estrogen receptor IHC staining from case #1. 100x

Three cases had high TMB, including 2 invasive ductal carcinomas and one invasive lobular carcinoma. All three cases showed high expression of ER but were negative for PR and HER2. All three cases showed prominent tumoral lymphocytic infiltrates (Fig. 2). Of these three cases, two harbored *MAGI2* mutations and one harbored a *PTEN* mutation. The two *MAGI2* mutations were MAGI2 S220* and MAGI2 Q1193fs*35. (Table 4).

**Table 4:** Three breast carcinoma cases with high TMB

Case	Age (yr)	Phenotype	ER	PR	HER2	Gene mutations	TMB (#/MB)
1	70	Lobular	95	0	Negative	*PIK3CA,****PTEN****, ARID1A, CDH1, CHD4, FAM123B, SMAD4, TP53*	33
2	50	Ductal, NOS	90	0	Negative	MAGI2*, PIK3C2B, PIK3CA, PIK3R1, SPEN, TP53	61
3	57	Ductal, NOS	99	0	Negative	*ERBB4, ESR1, GATA3, IGF1R,*MAGI2**, PAX5	20

Notes: *MAGI2 S220*; **MAGI2 Q1193fs*35

Abbreviations: *ER* estrogen receptor, *PR* progesterone receptor, *TMB* tumor mutation burden

## Discussion

Immunotherapy has demonstrated encouraging clinical benefits in advanced BC patients and PD-L1 IHC testing has been used to select eligible patients for such therapy [5]. However, challenges with current PD-L1 testing do exist, such as interassay variability and interobserver variability [11, 12]. Tumors with high TMB are associated with significant clinical benefit to immunotherapy in melanoma and non-small cell lung cancer patients [22, 27, 28]. TMB levels are very different among different tumors and such information is lacking in BCs [13]. In this study, we investigated TMB in 62 BCs determined by FoundationOne CDx assay and found a relatively low percentage of BCs with a high TMB level (3/62, 4.8%), consistent with previous study [13], but no association of TMB levels with any of the analyzed clinicopathologic characteristics was identified, such as age, histologic types and other biomarkers (ER, PR and HER2).

Tumors with deficient mismatch repair (dMMR) or microsatellite instability (MSI) have shown a high TMB level [13, 29–31] and patients with dMMR and MSI-high tumor have benefited from immunotherapy [32–35]. Tumors with DNA polymerase epsilon (POLE) mutation also have high TMB level [36]. While tumors with POLE mutation, dMMR, or high MSI show high TMB level, the reverse is not always true. For example, melanoma and non-small cell lung carcinomas frequently have high TMB but dMMR, MSI-high or POLE mutations are rare in these tumors [37–39], indicating other mechanisms can contribute to increased TMB [13, 32, 36, 40]. Previously, we and others have demonstrated the frequency of dMMR is very low in breast carcinomas [41–43]. In current study, significantly higher TMB was observed in breast cancers with DNA damage repair gene mutation(s) or *BRCA* (1/2) gene mutation, suggesting the importance of DNA damage repair proteins in maintaining DNA integrity and immune reaction. Tumors with DDR mutations generally represent a triple negative phenotype, higher tumor grade and other aggressive features. However, no such correlation was identified in tumors with DDR mutation. This would be caused by the low number of the cases tested in current cohort, thus, further studies with larger cohorts would be warranted.

Current cohort included two metaplastic carcinomas, one with a TMB value of 6 muts/Mb and the other one with a TMB value of 3 muts/Mb. Although the metaplastic carcinoma case number is very small, the findings of low TMB in these metaplastic carcinomas are consistent with a recent study which reported that the metaplastic carcinomas consistently expressed a low TMB of between 3 and 10 muts/Mb. In addition, two neuroendocrine carcinomas of our cohort also showed low TMB values, consistent with a previous study demonstrating neuroendocrine carcinomas of the breast tend to have low TMB [44].

In our study, three cases had high TMB and all showed prominent tumoral lymphocytic infiltrates, suggesting the association between TMB and immune reaction. Furthermore, of the three cases with high TMB, two harbored *MAGI2* mutations and one harbored a *PTEN* mutation. MAGI2 was initially characterized as a scaffolding protein that links cell adhesion molecules and receptors to cytoskeleton and maintains the architecture of cell junctions [45]. Further studies have revealed that MAGI2 promotes PTEN (tumor suppressor) function to regulate several kinase signaling pathways [46–48]. Additionally, MAGI2 is abnormally expressed in high grade prostatic intraepithelial neoplasia and prostate cancer compared to benign glandular epithelium [49–53]. In a recent study, glioblastoma patients who were not responsive to anti-PD-1 immunotherapy were significantly enriched for *PTEN* mutations and these *PTEN* mutations may induce a distinct immunosuppressive microenvironment, suggesting *PTEN* mutations’ involvement in immune reaction regualtion [54]. *PTEN* gene mutations are not uncommonly observed in many solid tumors, and are associated with immune suppression. In addition, loss of PTEN expression is correlated with up-regulation of PD-L1 in tumor cells and causes alteration in the tumor microenvironment, such as release of anti-inflammatory cytokines and significant reduction of T-cell activity [55]. Our findings of MAGI2 mutation in breast carcinoma with high TMB warrant future study to investigate MAGI2’s function in DNA repair pathway and PTEN signaling pathway.

Although this is one of the first studies to investigate TMB and its association with clinicopathologic features and genetic alterations in breast carcinomas, the significance of this study was limited by the small sample cohort (*n* = 62). The findings in current study need to be confirmed by future studies with larger cohort.

In conclusion, our data has demonstrated TMB levels were positively associated with TILs, but not any other analyzed clinicopathologic characteristics including breast cancer biomarker status, tumor histologic type and tumor nuclear grade. In addition, significantly higher TMB was observed in breast cancers with DNA damage repair gene mutation(s) or *BRCA* (1/2) gene mutation, suggesting the importance of DNA damage repair proteins in maintaining DNA integrity and immune reaction and breast cancer patients with DDR mutation may benefit from immunotherapy.

Ethics approval and consent to participate

All procedures performed in studies involving human participants were in accordance with the ethical standards of the institutional and/or national research committee.

## Abbreviations

PD-L1: Programmed death ligand 1
TMB: Tumor mutation burden
NGS: Next generation sequencing
BC: Breast cancer
ER: Estrogen receptor
PR: Progesterone receptor
HER2: Human epidermal growth factor receptor 2
IHC: Immunohistochemistry
DDR: DNA damage repair gene (DDR)
NER: Nucleotide excision repair
MMR: Mismatch repair
FA: Fanconi Anemia
HR: Homologous recombination
